# Data on customer perceptions on the role of celebrity endorsement on brand preference

**DOI:** 10.1016/j.dib.2018.03.138

**Published:** 2018-04-04

**Authors:** Ayodotun Stephen Ibidunni, Maxwell Ayodele Olokundun, Oyebisi Mary Ibidunni, Taiye Tairat Borishade, Hezekiah Olubusayo Falola, Odunayo Paul Salau, Augusta Bosede Amaihian, Peter Fred

**Affiliations:** a Department of Business Management, Covenant University, Ota, Ogun State, Nigeria; b Department of Accounting, Bells University of Technology, Ota, Ogun State, Nigeria

**Keywords:** Celebrity endorsement, Consumer brand preference, Brand association, Brand loyalty, Celebrity image

## Abstract

This research presents data on the effect of celebrity endorsement on consumers’ brand preference. Copies of structured questionnaire were administered to 384 customers of telecommunication industry. Using descriptive, correlation and regression statistical analysis, the data revealed that celebrity image has an effect on consumer brand loyalty, celebrity trustworthiness has an influence on consumer brand association. More so, the relationship between celebrity expertise and perceived quality of the product was established.

**Specifications Table**

**Table:** 

Subject area	*Management, Marketing Management*
More specific subject area	*Celebrity Endorsement, Brand Preference*
Type of data	*Table, figure*
How data was acquired	*Researcher made questionnaire analysis*
Data format	*Raw, analyzed, descriptive and statistical data*
Experimental factors	– *Samples consist of subscribers to mobile telecommunication services in Nigeria* – *In this paper, data evidence on customer perceptions on the role of celebrity endorsement on brand preference was presented.*
Experimental features	*Celebrity endorsement is a critical factor for enhancing customers’ brand preference*
Data source location	*Mobile telecommunication subscribers in Lagos State, Nigeria*
Data accessibility	*Data is included in this article*

**Value of the data**

• These data describe demographic data of mobile telecommunication customers in Nigeria and could be used by other researchers.
• The data describes celebrity endorsement as a strategic marketing communication programme that can enhance consumers’ preference towards a brand. Consequently, the data could provide insights for other researchers.
• The data allows other researchers to extend the statistical analysis.

## 1 Data

Table 1 represents demographic characteristics of respondents. The data showed 186 (51.2%) of the respondents were male and 177 (48.8%) were female. Also, the highest number of respondents were below 20 years (119(32.8%)).

**Table 1:** Demographic characteristics of respondents.

Parameter	Characteristics	Number (Percentage)
Gender	Male	186 (51.2)
	Female	177 (48.8)
Marital Status	Single	251 (69.1)
	Married	112 (30.9)
Age Bracket	Below 20	119 (32.8)
	21–30	131 (36.1)
	31–40	58 (16.0)
	41 and above	55 (15.2)
Educational Qualification	WAEC/O’level	160 (44.1)
	NCE/HND	34 (9.4)
	HND/BSC	105 (28.9)
	Postgraduate	64 (17.6)

Table 2 shows the regression and correlation relationships that exist between celebrity endorsement and consumer brand preference. Celebrity endorsement was measured using celebrity image, trustworthiness and expertise; consumer brand preference was measured using brand loyalty, brand association and perceived quality. Statistically, celebrity image was found to have a relationship with brand loyalty (r^2^ = 0.131; P ≤ 0.01). Trustworthiness was also revealed to influence on brand association (r^2^ = 0.208; P ≤ 0.01). Moreover, expertise was found to correlate with perceived quality (r^2^ = 0.432; P ≤ 0.01). The findings from this study is concurrent with findings from existing studies, such as [1], [2], [3], [4], [5], [11].

**Table 2:** Table 1: Data of dimensions of celebrity endorsement and brand preference.

**Variables**	**β**	**F-value**	**Sig.**	**P**	**R**^**2**^	**Statistical Analysis**
**Indep. → Dep.**						
Celebrity Image → Brand Loyalty	0.323	(1, 361) = 54.381	0.000	P ≤ 0.01	0.131***	Regression
Trustworthiness → Brand Association	0.368	(1, 361) = 94.826	0.000	P ≤ 0.01	0.208***	Regression
Expertise → Perceived Quality	–	–	0.000	P ≤ 0.01	0.432***	Correlation

*** p ≤0.01

## 2 Experimental design, materials and methods

Survey method was adopted to gather data. The population of this study are subscribers of telecommunication services in Lagos state Nigeria. The total population derived is 154,124,602 [6]. In order to determine the sample size, Godden [7] sample size formula was used. The reason for this choice of formula is that the population is infinite, that is the population greater than 50,000. Therefore, a sample size of 384 customers was determined for this study. Questionnaire was used to gather primary data from the respondents. This research benefitted from the ideas of existing research studies such as [8], [9], [10]. The collated data were coded and entered in SPSS version 22. Data analysis was performed applying descriptive, correlation and regression statistical test. Ethical consideration in the research process was ensured because administering the questionnaires to respondents was based on their willingness to respond to the research instrument. Moreover, confidentiality and anonymity for participants in the study was assured.
